# Challenges and Opportunities for Data Sharing Related to Artificial Intelligence Tools in Health Care in Low- and Middle-Income Countries: Systematic Review and Case Study From Thailand

**DOI:** 10.2196/58338

**Published:** 2025-02-04

**Authors:** Aprajita Kaushik, Capucine Barcellona, Nikita Kanumoory Mandyam, Si Ying Tan, Jasper Tromp

**Affiliations:** 1 Saw Swee Hock School of Public Health National University of Singapore Singapore Singapore; 2 Duke-NUS Medical School Singapore Singapore

**Keywords:** artificial intelligence, data sharing, health care, low- and middle-income countries, AI tools, systematic review, case study, Thailand, computing machinery, academic experts, technology developers, health care providers, internet connectivity, data systems, low health data literacy, cybersecurity, standardized data formats, AI development, PRISMA

## Abstract

**Background:**

Health care systems in low- and middle-income countries (LMICs) can greatly benefit from artificial intelligence (AI) interventions in various use cases such as diagnostics, treatment, and public health monitoring but face significant challenges in sharing data for developing and deploying AI in health care.

**Objective:**

This study aimed to identify barriers and enablers to data sharing for AI in health care in LMICs and to test the relevance of these in a local context.

**Methods:**

First, we conducted a systematic literature search using PubMed, SCOPUS, Embase, Web of Science, and ACM using controlled vocabulary. Primary research studies, perspectives, policy landscape analyses, and commentaries performed in or involving an LMIC context were included. Studies that lacked a clear connection to health information exchange systems or were not reported in English were excluded from the review. Two reviewers independently screened titles and abstracts of the included articles and critically appraised each study. All identified barriers and enablers were classified according to 7 categories as per the predefined framework—*technical*, *motivational, economic*, *political*, *legal and policy*, *ethical*, *social*, *organisational*, and *managerial*. Second, we tested the local relevance of barriers and enablers in Thailand through stakeholder interviews with 15 academic experts, technology developers, regulators, policy makers, and health care providers. The interviewers took notes and analyzed data using framework analysis. Coding procedures were standardized to enhance the reliability of our approach. Coded data were reverified and themes were readjusted where necessary to avoid researcher bias.

**Results:**

We identified 22 studies, the majority of which were conducted across Africa (n=12, 55%) and Asia (n=6, 27%). The most important data-sharing challenges were unreliable internet connectivity, lack of equipment, poor staff and management motivation, uneven resource distribution, and ethical concerns. Possible solutions included improving IT infrastructure, enhancing funding, introducing user-friendly software, and incentivizing health care organizations and personnel to share data for AI-related tools. In Thailand, inconsistent data systems, limited staff time, low health data literacy, complex and unclear policies, and cybersecurity issues were important data-sharing challenges. Key solutions included building a conducive digital ecosystem—having shared data input platforms for health facilities to ensure data uniformity and to develop easy-to-understand consent forms, having standardized guidelines for data sharing, and having compensation policies for data breach victims.

**Conclusions:**

Although AI in LMICs has the potential to overcome health inequalities, these countries face technical, political, legal, policy, and organizational barriers to sharing data, which impede effective AI development and deployment. When tested in a local context, most of these barriers were relevant. Although our findings might not be generalizable to other contexts, this study can be used by LMICs as a framework to identify barriers and strengths within their health care systems and devise localized solutions for enhanced data sharing.

**Trial Registration:**

PROSPERO CRD42022360644; https://www.crd.york.ac.uk/prospero/display_record.php?RecordID=360644

## Introduction

### Overview

Artificial intelligence (AI) has the potential to revolutionize health care by empowering health care practitioners in supporting clinical decision-making, enhancing diagnostic accuracy, and refining administrative processes for health care providers, payers, and pharmaceutical organizations [1-5]. Applications such as AI-driven predictive analysis and personalized treatments can improve health care delivery, even in low- and middle-income countries (LMICs). However, despite the promising aspects of AI in health care, there are significant challenges related to limited data sharing and accessibility that impede the development and localization of AI applications in health care, particularly in LMICs [1-3,6-9].

### Rationale

Previous studies have identified challenges to health information exchange (HIE) and data sharing, more generally [10-14]. Akhlaq et al [10] reviewed barriers and facilitators to implementing and adopting HIE. This systematic review identified 7 themes influencing HIE—sociopolitical, financial, infrastructure, organizational, technical, individual, and data management [10]. Similarly, Figueiredo [11] and van Panhuis et al [13] identified 20 potential barriers to data sharing across 6 domains—technical, motivational, economic, political, legal, and ethical. However, gaps exist in current research. First, these studies did not focus specifically on the granular and often sensitive data required for AI models in health care, such as high-quality clinical datasets and high-resolution medical images. The complexity of these data types introduces additional challenges, such as ensuring data interoperability across different systems and institutions, addressing heightened privacy concerns, and maintaining data quality for AI algorithms. Furthermore, current studies do not fully test these AI-specific barriers. Second, the absence of empirical testing of frameworks using in-depth formative research is also a pressing gap across current literature. Engaging stakeholders (eg, policy makers, health care providers, and data scientists) in LMICs is critical for identifying practical solutions and for refining theoretical frameworks to fit real-world contexts. Defining clear metrics for the success of data sharing, for example, improved interoperability, increased data accessibility, and reduced privacy risks within national and organizational frameworks, not only informs scalable, adaptable solutions for AI in LMICs but also allows for their evaluation [15].

### Thailand

Thailand is developing its AI capabilities and promoting AI adoption, but significant obstacles remain, particularly regarding data sharing. The fragmented nature of its health care service landscape and unclear data-sharing guidelines restrict the effective use of AI in health care. The Thailand National AI Strategy and Action Plan (2022-2027) attempts to close this gap by establishing a data-sharing guideline to enable AI deployment [16]. According to the Government AI Readiness Index 2021, Thailand ranked 59th globally and 9th in East Asia, demonstrating moderate progress in AI readiness but highlighting areas that require improvement, especially in health data governance [17,18].

### Aims

This study aimed to address the research gaps by conducting a systematic review to identify the barriers and enablers to data sharing for AI in LMICs. The study further builds on existing literature by testing these findings through stakeholder interviews in Thailand—collecting empirical evidence on local challenges and enablers for AI-related data sharing and future recommendations for sharing data related to AI tools in Thailand. This mixed methods approach allows us to capture both the broader trends from LMICs and context-specific insights from Thailand, contributing to a more nuanced understanding of the intersection between AI and health care data sharing. Our findings will inform policy makers, health care organizations, and technology developers on building effective AI data-sharing frameworks, closing the gap between policy and implementation.

## Methods

### Overview

We conducted the study in 2 phases. First, we performed a systematic search to identify the literature assessing barriers and facilitators to sharing data for AI in health care. We synthesized and categorized findings using a predefined framework. Second, we conducted semistructured qualitative stakeholder interviews, which we analyzed thematically using the framework’s domains. The qualitative interviews’ findings validate, complement, and reiterate findings from the systematic review to a localized context (ie, Thailand) and supplement possible gaps in the framework.

### Framework

We devised a predefined framework to categorize barriers and enablers on the basis of 2 previously published frameworks for data sharing [13,19]. The framework included 7 domains categorizing barriers and enablers to data sharing: technical; motivational; economic; political, legal, and policy; ethical; social; organizational, and managerial. These categories are defined in detail in Textbox 1.

**Classification of factors of data sharing and their definitions.**

- Technical: barriers and enablers relating to health information system capacity and other technological tools
- Motivational: barriers and enablers relating to personal or institutional motivations to share data
- Economic: barriers and enablers relating to the financial sustainability and costs of data sharing
- Political, legal, and policy: barriers and enablers relating to political principles, regulations, and public policy
- Ethical: barriers and enablers relating to moral principles, values, and ethical guidelines
- Social: barriers and enablers relating to societal norms and attitudes toward data sharing and health technology
- Organizational and managerial: barriers and enablers relating to organizations’ strategy, leadership, and management practices

### Systematic Review

#### Search Strategy and Inclusion and Exclusion Criteria

We used the SPICE (settings, perspective, intervention, comparison, and evaluation) framework (Textbox 2) to inform our search strategy and inclusion and exclusion criteria. The selection process focused on studies that explored AI tools, machine learning (ML), electronic decision support systems, or digital interventions, specifically in the context of HIE. We included primary research studies (empirical studies investigating the development, implementation, or evaluation of digital health tools related to HIE); perspectives (opinion or viewpoint articles offering expert insights into AI, ML, electronic decision support systems, or HIE systems); policy landscape analyses (reviews examining the policy or regulatory environment around digital health and HIE); and commentaries (discussions of challenges, advancements, or ethical considerations related to the use of digital interventions in HIE). Only studies published until October 2022 were considered. The inclusion criteria required studies to have a specific focus on the technical and practical aspects of HIE, such as how AI or ML tools facilitate data exchange, the role of interoperability, or the establishment of secure access protocols. Articles that did not address these core components or lacked a clear connection to HIE systems were excluded from the review. All included studies had to be performed in or involve an LMIC context, according to the World Bank country classification (fiscal year 2019) [20]. We excluded studies not reported in English. We defined AI in health care as any software or algorithm made to automate intelligent behavior in a health care setting for diagnosis or treatment directed toward patients, caregivers, health care providers, or a combination [21]. We registered the review protocol with PROSPERO before commencing data extraction (CRD42022343604).

We systematically searched PubMed, SCOPUS, Embase, Web of Science, and ACM. In the search strategy, we incorporated keywords and controlled vocabulary terms for the key concepts of data sharing, AI tools, health care, and barriers and enablers (Multimedia Appendix 1). A librarian at the National University of Singapore vetted the strategy.

**The SPICE (settings, perspective, intervention, comparison, and evaluation) framework for conducting the systematic review.**

**S (settings)**

- Health care domain

**P (perspective)**

- Experiences, perspectives related to barriers and enablers to data sharing

**I (intervention)**

- Artificial intelligence tools, machine learning, digital health, electronic decision support systems

**C (comparison)**

- Traditional health setting or not applicable

**E (evaluation)**

- Challenges and barriers to data sharing
- Facilitators and enablers to data sharing
- Identification of groups or stakeholders and their perspectives

#### Data Synthesis

The retrieved search results were imported to Clarivate Endnote (version 20.3) and deduplicated. The remaining articles were imported to Rayyan. Two reviewers independently screened the titles and abstracts of the included articles. All articles that potentially met the inclusion criteria and were agreed upon by both reviewers were included in the review for full-text screening. A third reviewer resolved conflicts.

Data extraction tools were developed, and the data were synthesized. These included article title, first author, year of publication, study characteristics—including country of origin, study design (qualitative and semistructured), description of AI tool, and a summary of barriers and enablers to data sharing. Data synthesis was on the basis of The Joanna Briggs Institute’s (JBI) meta-aggregative approach for qualitative systematic reviews [22]. The qualitative data were pooled and represented as a narrative summary for each study per domain.

#### Critical Appraisal

Two reviewers (AK and CB) critically appraised each study independently using the Critical Appraisal Skills Programme checklist for qualitative studies (Multimedia Appendix 2) [1-3,6-9,23-36] [37]. The Critical Appraisal Skills Programme checklist was used because it is appropriate for all types of qualitative research (including landscape analysis and perspectives).

### Stakeholder Interviews

We tested the relevance of barriers and enablers using Thailand as a case study. Thailand was chosen because of the publication of its recent National AI Strategy and Action Plan (2022-2027) and because coauthors of this study had access to local stakeholders [16]. We conducted an exploratory qualitative study to better understand the various challenges faced by the stakeholders in data sharing for AI and the facilitators that help overcome this at a local level.

### Study Design

We identified and mapped potentially relevant stakeholders through social media (LinkedIn), search engines, personal, company (university and ministry), and official websites. We included stakeholders aged ≥21 years who could communicate verbally in English, academic experts, technology developers, regulators, or policy makers in governance, regulation, and implementation of digital health technology and novel technology in Thai health care. We used purposive and snowball sampling techniques for stakeholders until saturation was reached.

All mapped stakeholders were contacted via an email titled “Invitation to discuss data sharing for artificial intelligence in Thailand” that included the semistructured interview guide (Multimedia Appendix 3) and the standard letter of invite entailing a short summary of the project, information for the participants regarding the type of data collected, and how data will be used. This aimed to ensure participants could make informed decisions about which interview questions they preferred to answer.

All 5 authors conducted stakeholder interviews on Zoom from December 20, 2022, to February 10, 2023. Interviews followed a semistructured interview guide. The interview guide was vetted by all authors and included relevant comments as per each author’s expertise and was further revetted by our partners at the Office of National Higher Education Science Research and Innovation Policy Council, Thailand, to test the local relevance of the topics included and to avoid any potential bias.

### Data Collection

Interviewers took detailed written notes, which were subsequently reviewed for accuracy and completeness. The notes were then coded based on predefined domains of barriers and facilitators aligned with the framework. Two researchers independently coded the data using MS Excel, systematically comparing coded segments and themes across interviewees. Illustrative, nonattributable quotations supported the findings, providing a rich, contextual understanding of the data. The coded data were reverified during the analysis, and themes were readjusted where necessary with consensus to avoid researcher bias.

### Data Analysis

We conducted a thematic analysis using framework analysis to qualitatively analyze the interview data. This approach is well-suited for applied policy research, as it systematically explores stakeholders’ views, knowledge, and perspectives while retaining the contextual integrity of individual narratives. The framework analysis method involves 5 distinct stages: familiarization, identifying a thematic framework, indexing, charting, and mapping and interpretation, which provides a structured approach to organizing and interpreting the data [38].

Throughout the analysis, reflexivity was emphasized, as the researchers continuously examined their own perspectives and potential biases by engaging in discussions to ensure that personal assumptions did not influence the interpretation of the data. We addressed the 3 key criteria for ensuring trustworthiness in qualitative research. (1) Confirmability: we documented the coding and thematic development processes and conducted consensus meetings between coders to ensure that the findings were grounded in the data rather than influenced by researcher bias. (2) Dependability: to enhance the reliability of our approach, we standardized the coding procedures. Consistent data management practices were followed, and any discrepancies between coders were resolved through discussion. (3) Credibility: illustrative, nonattributable quotations supported the themes and ensured that the findings accurately reflected the participants’ views.

### Ethical Considerations

The National University of Singapore Institutional Review Board approved the qualitative component of this study (NUS-IRB-2022-550). No interviews were recorded, and consent was obtained via email and at the start of the interview. The interview data were not anonymized and were retained solely for potential future contact regarding the study. Participants were informed and consented that their personal information (limited to name, email address, and designation) would be securely stored according to the National University of Singapore’s data storage principles and policies, accessible only to the project team. Consent was obtained to use select quotes from the interviews, ensuring that no identification of individual participants is possible. No compensation in any form was provided to participants.

## Results

### Systematic Review

#### Study Characteristics

**Figure 1:**
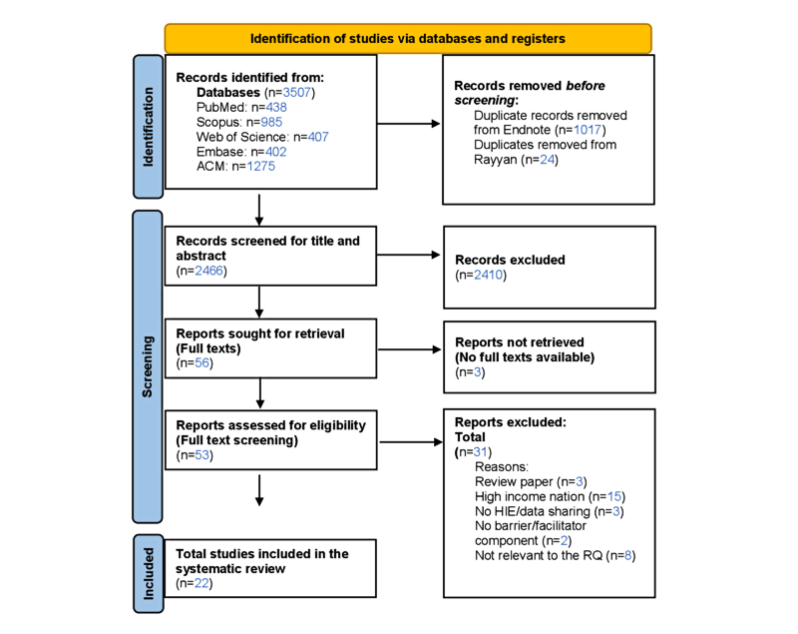
PRISMA (Preferred Reporting Items for Systematic Reviews and Meta-Analyses) flow diagram for included studies. HIE: health information exchange; RQ: research question.

We retrieved 3512 records, with 2471 articles left after removing duplicates (Figure 1). After excluding titles and abstracts, 58 articles were eligible for full-text screening. Finally, the data extraction and qualitative synthesis included 22 articles published between January 2010 and October 2022 that fulfilled the inclusion criteria.

#### Quality Appraisal

Table S1 in Multimedia Appendix 2 presents the quality appraisal results. All studies reported a clear statement of the aims and findings of the research. The qualitative methodology was appropriate to all studies, while the research design was appropriate to all except one [3]. The appropriateness of recruitment strategy and data collection was unclear or inappropriate for 7 [2,3,23-26,39] and 6 studies [2,3,23,25-27], respectively. Whether the relationship between researchers and participants or ethical issues was adequately considered was unclear or not appropriate for 11 [1,2,6,23,24,26-31] and 12 studies [1,6,23,24,26-28,30,32-34], respectively. All studies were valuable to the local context.

Table 1 shows the characteristics of the included studies. Of all the studies, 8 (36%) were landscape analyses, 7 (32%) had a qualitative design, 2 (9%) were mixed methods, 3 (14%) were case studies, and 2 (9%) were perspective articles. Of all the studies, 12 (55%) studies were from Africa, 6 (27%) from Asia, and 2 (9%) from Latin America and the Caribbean.

**Table 1:** Characteristics and description of included studies.

Study	Country	Study design	AI^a^ tool
Akhlaq et al [35], 2020	Pakistan	Exploratory mixed methods case study	HIE^b^ in Pakistan
Bhattacharya and Pandey [23], 2021	Global	Analysis	Internet of things
Botha et al [7], 2015	South Africa	Qual-quant mixed methods analysis with stakeholder interviews and a survey	eHealth systems in South Africa
Chali et al [2], 2017	Tanzania	Case study	Mobile DEC^c^ for Tanzanian EHRs^d^
Chukwu et al [1], 2022	Sierra Leone	Semiqualitative primary study	Digital health solutions eIDSR^e^ and DHIS2^f^, data sharing practices, and current data use in Sierra Leone
de Fatima Marin et al [28], 2022	Brazil	Digital health landscape analysis	National Health Data Network (RNDS^g^)
deRiel et al [31], 2018	Haiti	Analysis	EMR^h^ program
Fakhkhari et al [6], 2019	Morocco	Analysis	EHR in public hospitals inside and outside Morocco
Guidry et al [8], 2010	Kenya	Information systems landscape analysis	Data models and information systems in Kenya for HIV care
Keny et al [33], 2015	Kenya	Primary qualitative case study	EHR in Kenya
Lei et al [24], 2017	China	Primary qualitative case study	HIE in Xinjin
Muinga et al [36], 2020	Kenya	Semistructured qualitative study	Digital health systems in Kenyan public hospitals
Ndlovu et al [25], 2016	Botswana	Landscape analysis	ICTs^i^ in Botswana
Ntlhakana et al [29], 2022	South Africa	Semiqualitative cross-sectional study	South African mines’ HCPs^j^ data
Nutley et al [34], 2013	Kenya	Semistructured qualitative study	DHP^k^ tool in Kenya
Pradhan et al [3], 2021	India	Digital health landscape analysis	Overview of AI in health care
Shrivastava and Srikanth [26], 2020	India	Case study	Federated digital health care ecosystem in India
Simbini et al [39], 2018	Africa	Perspective	African eHealth strategies and ICT
Troncoso [27], 2020	LMICs^l^	Perspective	AI and ML^m^ in primary health care
Walcott-Bryant et al [32], 2021	Kenya	Primary qualitative case study	Digital health solution to support management of hypertension in Kenyan private health sector
Wang et al [30], 2019	China	Case study without stakeholder interviews	EMR data
Yaqoob et al [9], 2017	Pakistan	Feasibility analysis	EHR and NHIS^n^ in Pakistan and other countries

^a^AI: artificial intelligence.

^b^HIE: health information exchange.

^c^DEC: Data Exchange Component.

^d^EHR: electronic health record.

^e^eIDSR: electronic Integrated Disease Surveillance.

^f^DHIS2: District Health Information Software.

^g^RNDS: National Health Data Network.

^h^EMR: electronic medical record.

^i^ICT: information and communications technology.

^j^HCP: hearing conservation program.

^k^DHP: District Health Profile.

^l^LMIC: low- and middle-income country.

^m^ML: machine learning.

^n^NHIS: National Healthcare Information System.

#### Factors Associated With Data Sharing

**Figure 2:**
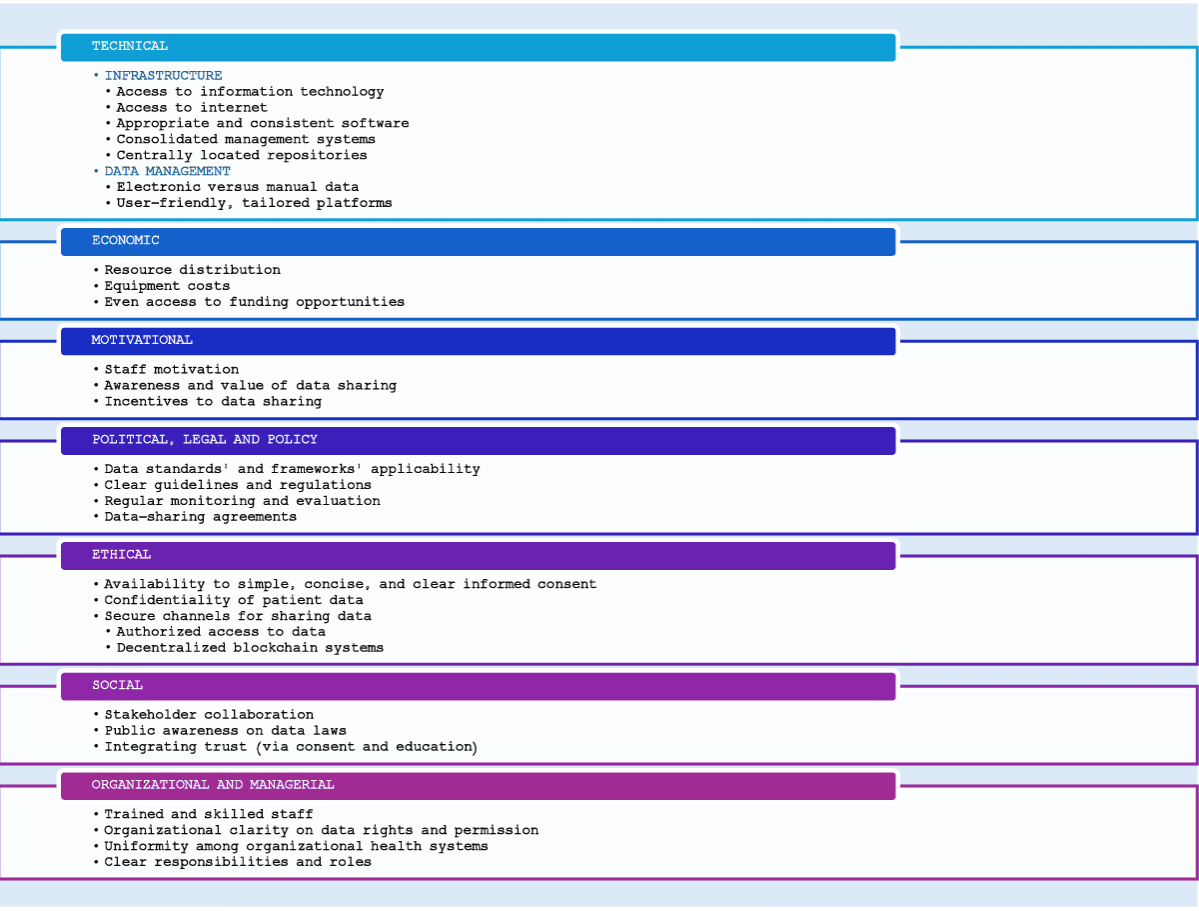
Factors associated with sharing of health care data using artificial intelligence tools.

Figure 2 summarizes the factors associated with data sharing according to the framework.

##### Technical Factors

We identified 7 technical factors associated with data sharing for AI in LMICs; 5 were related to infrastructure, and 2 were related to data management practices. Limited access to information and communications technology and poor infrastructure lead to poor internet connectivity, power shortages, and software crashes, causing difficulty in backing up and exchanging data [7,23,28,35,36]. Inappropriate and inconsistent data management software and software versions can lead to data fragmentation and format issues [2,29]. Health systems in LMICs often neither use consolidated data management systems (ie, a system that seamlessly brings together various subsystems into one for easier management and coordination [40]) nor use central repositories (ie, a singular storage location for data within an organization, providing visibility, collaboration, and consistency within data management [41,42]). HIEs are often standalone [8,9,35] with limited communication across different databases and various health systems and electronic medical record (EMR) systems implemented in different districts or provinces, leading to duplication [25,32]. This can lead to inconsistent use of data definitions and labels, reducing interoperability [2,8,33]. Investing in a multisourced data triangulation system, storing data in a single database and central repository, and using standardized electronic health records (EHRs) can promote data collection and reduce fragmentation [1,6,9,28-30,35].

Regarding data management, many health care systems in LMICs use handwritten records, often in native (ie, regional) languages [26,34], limiting shareability. Ensuring that the platforms and software used for data handling are ergonomic, user friendly, and tailored to local and regional contexts will increase their uptake and use among health care staff [6].

##### Motivational Factors

We identified 3 motivational factors. First, health care staff often lack the motivation to systematically collect data and adopt new standards for uniform data collection [8,33]. Education and awareness about the value of health data exchange can address staff resistance to digital data collection [24,27]. Second, managers and organizations might not perceive the value of collecting health care data or emphasize the importance of data quality to their staff [8,43]. Third, incentives for adopting and using AI can improve the digitization of medical data [24,27].

##### Economic Factors

We identified 3 economic factors associated with data sharing. First, the uneven distribution of financial, human, and technical resources in LMICs [24,35] is a significant barrier for small clinics or hospitals to collect high-quality data [7,9]. Limited resources constrain the use of data audits for confidentiality, privacy, and standardized data field terminologies [25,31,33]. High equipment costs are a significant barrier for small clinics or hospitals to collect. Financial support enables the adoption of EMR systems and promotes the implementation of regional HIEs by addressing infrastructural barriers, lack of human resources, adequate training, data quality, and security challenges [7,24,35].

##### Political, Legal, and Policy Factors

We identified 4 political, legal, and policy factors. First, transparent regulations, defined standards for intersystem communication, anonymization of personal data, and security frameworks promote data sharing [3,9,23,27,30,39]. Second, national policy and legal facilitators for data sharing include a national strategy [24,33,35] and data-sharing guidelines to clarify national personal data protection acts [2,3,32]. However, even when national data policies are in place, ensuring regulatory compliance can be challenging [7,24,25]. Data auditing mechanisms can promote compliance [30,39]. Finally, clear institutional-level data-sharing guidelines promote information transfer and increase data-sharing motivation. Similarly, having confidentiality agreements helps uphold data accuracy and privacy protection requirements [25,30,31]. Aligning the description of data elements with current clinical practices and workflows will facilitate work for health care staff and enhance collaboration among entities involved in health data sharing [39].

##### Ethical Factors

We identified 3 ethical factors. The absence of explicit informed consent and patient confidentiality concerns are significant data-sharing barriers, while unauthorized access caused by bypassing security mechanisms can cause ethical concerns, further putting the patient’s data in jeopardy and thus creating a reluctance to share data among patients and practitioners [3,9,26]. Frameworks focusing on ethics and privacy at the national and institutional level facilitate trust among data users and patients [27]. Creating secure channels for data sharing and state-of-the-art security standards will also be important to securing patient privacy [23,31]. Novel technologies such as decentralized consent management blockchain systems might be a potential solution, enabling patients to share consented data through data requests [32].

##### Social Factors

We identified 3 social factors associated with data sharing. First, the complexity of the types of stakeholders involved in data sharing limits collaboration and interoperability [2,3]. Second, a lack of public awareness regarding patient rights and data laws can also be a barrier that may hinder consent processes [26]. Third, national protocols, certification systems, and increased awareness and information about the benefits and risks associated with data sharing will help build trust between health care practitioners and patients [3,27,30].

##### Organizational and Managerial Factors

There were 4 organizational and management factors. Workforce limitations [12,13], limited provider digital literacy [24,25,32,35,36,43], and the absence of expert IT teams [6] reduce data capture and quality. Overburdened health care workers’ conflicting priorities limit the time to enter high-quality data into digital systems [36,43]. A shortage of staff and leaders can lead to ill-defined responsibilities and ambiguity of data-related roles, further enhancing the ineffectiveness of organizational procedures [7,29,33]. Poor organizational clarity on data rights and permissions, central coordination, and terminology are barriers to data sharing [9]. This can be addressed using standard operating procedures to facilitate health data exchange across facilities [1,29]. Health personnel training can enable the staff to use EMR systems, manage data dictionaries, address data quality challenges, and address reluctance to use digital technology [7,24,31,33,35,43]. The complexity of stakeholders and multiple system contractors and organizations involved in digital health in developing nations is a significant barrier to interoperability [11].

### Stakeholder Interviews

We mapped 50 relevant stakeholders for interviews, of which 15 confirmed participation (response rate: 15/50, 30%). Most interviewees were academic experts (6/15, 40%) and technology developers (5/15, 33%). The remaining were policy makers, regulators, and health care experts. Table 2 summarizes the major themes from the interviews. We identified multiple barriers and enablers relevant to the context of Thailand, which were classified under the 7 main domains. Most stakeholders reiterated the technical, economic, political, and social factors of data sharing identified during the systematic review.

Thematic synthesis of the interviews yielded multiple barriers and enablers relevant to Thailand’s context, which were classified under 7 main categories of our framework.

**Table 2:** Summary of major themes identified through in-depth stakeholder interviews.

Framework	Subthemes identified by stakeholders
Technical factors	Inconsistent data architecture, formats, and standards, eg, different data formats between different EHR_a_ platforms of the same organization Misalignment in terminology and structure and data inaccuracy Different data collection practices between private and public health care sectors Manual data management and paper-based EHRs
Motivational factors	Lack of staff time General unawareness of the value of sharing data, creating awareness and empowerment Limited incentives to sharing data beyond the organization; therefore, providing incentives such as tax rebates and funding to private sectors
Economic factors	Lack of financial support and resources (mainly manpower and trained staff) High equipment costs Financial disparity between private and public sectors increases the gap in digital skills proficiency
Political, legal, and policy factors	Complex or unclear data-sharing regulations and guidelines (eg, what data can be shared and what needs to be protected) between organizations or hospitals cause fear of sharing data wrongly Too many laws and types of laws cause confusion, making data integration cumbersome Low applicability of international standards Having clear guidelines mandating standardized practicing on data entry, data protection, and data storage Identifying a single government organization responsible for collaborating and strengthening all branches of data sharing
Ethical factors	Confidentiality and consent issues Frequent breaches of hospital information systems Improving adherence to practices that mitigate data breaches, such as separating personal and professional use of computers, using 2-factor authentication or a password-protected log-in
Social factors	Lack of public awareness of data laws and low digital health literacy Limited stakeholder collaboration
Organizational and managerial factors	Lack of clarity on what data can be shared Issue of data ownership, ie, considering data as private and profitable assets Lack of an organizational protocol, ie, standard operating procedures with necessary steps to approve data-sharing, data protection, and staff training guidelines

^a^EHR: electronic health record.

#### Factors Associated With Data Sharing: Thematic Analysis

##### Technical Factors

Stakeholders reported that current data architecture and inconsistent data standards (multiple or uneven systems) did not allow hospitals to share data throughout the health care system. There can be a lack of software standardization even within the same company. In addition, manual data management and paper-based EHRs are common. In lower-income regions or smaller clinics, there is a lack of equipment and infrastructure to collect data (and share data safely), leading to uneven and fragmented data. One participant mentioned that different data collection and health information systems in the clinical workflow are barriers to efficiently sharing data. Most participants also noted the lack of uniformity in gathering data across private and public health sectors:

In some clinics, their systems are not fully electronic yet (e.g., patient hand writes, then the document is scanned into a PDF form and is uploaded).

Policy expert

Previously, governments had no standards for sharing data, which led to each hospital having their own system, format, and structure.

Health care expert

There is neither a centralised system nor initiative to streamline all the healthcare data into a single platform like a National EMR system.

Academic expert

To overcome these technical barriers, stakeholders emphasized the need for a decentralized data infrastructure system to enable data sharing. This can be achieved by making routine data collection electronic, keeping in mind its user friendliness, which would not exacerbate staff workload:

All healthcare facilities need to use one single platform for data input in the future. This is important to ensure comparability of the data.

Academic expert

##### Economic Factors

Stakeholders reported a lack of funding and resources (mainly manpower and staff time) as a barrier to standardization, scaling, and data sharing. They cited high equipment and software costs as a reason for still using paper-based data collection. Funding inequity caused the disparity in digital skills between low versus high-income facilities. Stakeholders mentioned that regular top official turnover limited consistent IT staff training. All these barriers led to a lack of technical capacity to digitize patients’ records, especially in most rural hospitals:

There is a lack of equity (in funding)... In low-income health facilities, the manpower may not have the technical capabilities to update data and link the data to a central database.

Policy expert

...The public sector in Thailand should work on security, but it is beyond their budget to buy large tools such as firewalls.

Academic expert

... unclear who would bear the cost of operating a unifying health data platform in the future.

Academic expert

None of the stakeholders mentioned any solutions to the aforementioned economic barriers that would help facilitate data sharing.

##### Political Factors

Most reported political- and policy-level barriers revolved around the lack of data-sharing regulations and guidelines (eg, who can share what data and under what circumstances), complex and unclear policies, and the low applicability of international data standards. Participants mentioned that there were no clear regulations and national policies on data sharing between hospitals, which may cause the doctors to reject patient requests to share their information with another hospital. Declining requests to share data was also attributed to a fear caused by the lack of clear laws: no laws would protect and support hospitals if they wrongly released a patient’s health data. Therefore, too many laws or types of laws were reported to cause confusion and make data integration even more difficult. Other reported barriers were the lack of cross-ministry collaboration and the absence of a central regulatory authority that modulates data-sharing practices among health care providers:

The Ministry of Health should work with other ministries to develop these (data sharing guidelines).

Technology developer

Current law (law that governs digital platforms) is not enough to cover digital health services; an extension of the law is required.

Regulator

Possible solutions to these political-level barriers ranged from government decisions to standardize data inputs and sharing to using digital health sandboxes. It was suggested that data sharing between data collectors should be mandated by law, that regulations should be implemented, that there should be an intermediary between all ministries to facilitate collaboration, and that there should be a national committee on AI in health care with a high priority for data sharing:

It is suggested that both the public and private sector agree on a set of standards so that they are willing to share the data collected nationally.

Technology developer and policy expert

Steer the development of regulatory sandboxes as testbeds for regulatory innovation ... it is also important to provide clear costs and benefits for start-ups to join the sandboxes right at the beginning.

Technology developer

##### Social Factors

Important social barriers included a lack of awareness of data laws, data sharing, and collaboration. Hospitals often feel reluctant to share data with private companies because of limited awareness of laws on data sharing (eg, Personal Data Protection Act [PDPA]). Perceived gray areas and limited regulatory clarity exacerbate unclarity on who can share what data under which circumstances. Another significant barrier included the absence of a central body that overlooks communication between different ministries to facilitate interministerial collaboration and data transfer:

The implementation of PDPA was launched in Thailand and people stopped sharing data altogether. This is clear evidence that the people have misunderstood the meaning of data sharing.

Policy maker

There is poor collaboration between the public and private sector; limited evidence and experience related to data sharing.

Academic expert

In response to these barriers, interviewees suggested collecting input from various stakeholders involved in data sharing. These should include individuals in the medical field, companies, ministries, and patient organizations. Identifying a single government organization responsible for collaborating and strengthening all branches of data sharing is an unmet need. Another critical consideration was enhancing the Thai population’s awareness and knowledge of data sharing:

Various stakeholders will need to come together to work together in building a conducive digital ecosystem that facilitates responsible data sharing.

Academic expert

##### Motivational Factors

One participant reported a lack of staff time as a barrier. Other motivational barriers included people’s (eg, patients, health care providers, and health staff) unawareness of the value of sharing data and limited incentives for organizations to share data:

Doctors often lack the motivation to share data as they dislike sharing data amongst themselves, as they do not like to share their logic and treatment process for a patient.

Health care expert

Public and private healthcare facilities are unlikely to pull resources and share their data across the public and private spheres.

Academic expert

Creating incentive structures for hospitals to share data was among the most widely suggested enablers. One participant suggested that the government can consider providing funding and tax incentives to the private sector if they share data for the public good. Regarding creating awareness about data sharing and data laws, interviewees suggested that executive-level players in organizations, entrepreneurs, and policy makers must be empowered and educated. Patients should receive information on the benefits of sharing their data. Furthermore, data owners should have an understandable and unambiguous process to share their data securely:

Doctors currently do not have the mindset to share the data that they collect. Therefore, it is suggested that there should be incentives from sharing data, such that the one that collects the data (medical doctors) as well as the individual that analyses the data both benefits.

Policy expert

For instance, an application to use safe drugs would encourage hospitals and drug stores to provide information directly to patients. The inventive for these pharmacies would be patient engagement; there is a greater demand and therefore greater profit which acts as an incentive to the pharmacy. This would allow the information of the individual and the history of purchases to be recorded—whether in the form of websites or memberships—which would encourage customers to come back and buy from the same drug store.

Technology developer and policy expert

Increase the awareness of data owners; create simpler consent form that is clear, concise and explicit.

Technology developer

##### Ethical Factors

Ethical barriers included a lack of awareness of data laws (eg, PDPA) and data privacy and security issues:

Patients are afraid of sharing their data because of the increased rate of cyberattacks on hospitals.

Policy expert

Data security discipline among users can mitigate data breaches and help overcome ethical barriers. Possible solutions include separating personal and professional computer use, using 2-factor authentication, and using password-protected log-in and log out:

To enable responsible data sharing in digital health, cybersecurity of the health information systems will have to be strengthened. The country will also need to come up with a clear structure to compensate victims whose data are compromised.

Academic expert

##### Organizational and Managerial Factors

Interviewees reported that there are regulations whereby management teams are unable to ensure whether data are secure and safe enough to share, which acts as a barrier at the organizational level (ie, there are certain workflows and policies that fall within a gray area, as mentioned previously). Bureaucracy was also mentioned as a barrier if considering the top-down approach, as changes in high-level leadership in the ministries and organizations caused delayed processing and changed regulations related to data sharing. Data ownership was reported to be a disputed area where health care providers and private hospitals were unwilling to share the collected data, considering it an asset for business profitability. The managerial mindset of organizations regarding data ownership, that is, the notion that if they have generated the data, the data belongs to them, causes unwillingness to share data with other organizations:

Mindset of care providers that just because the patients are willing to share their data, the data is now owned by the hospital. However, this is untrue as the hospitals are simply data collectors, and fundamentally, the Personal Data Protection Act (PDPA) states that the personal health information belongs to the patient.

Technology developer and policy expert

Different organisations (and even departments in the same organisation) do not want to share data as they believe that it is valuable and profitable.

Academic expert

Only a few interviewees provided solutions to some organizational barriers. One suggested that having an organizational protocol with the necessary steps to approve data sharing would enhance structure and increase trust:

Hospital administrators need to be trained on cybersecurity safeguards, data security, and be given access to resources to improve their digital infrastructures to enable digital transformation (i.e., transforming paper-based data to machine readable data).

Academic expert

## Discussion

### Principal Findings

Our systematic review identified 29 unique factors across the 7 domains associated with data sharing for AI in LMICs. These factors can significantly influence the ability of health care organizations to share data with AI developers and limit the implementation of AI solutions in health systems. Our case study in Thailand demonstrated the relevance of the domains, serving as an example of how the factors identified in the systematic review can be contextualized locally. Strategies to address barriers to data sharing in countries with nascent AI industries can consider the factors identified in this systematic review for policy development.

Two previous studies identified barriers to HIE adoption in LMICs [10] and sharing research and scientific data [11]. Our review is distinct from this previous work by focusing on factors associated with data sharing for AI in health care. Data used to develop AI require more granularity (eg, in the form of text or images). This influences the factors associated with data sharing. For example, data used to deploy AI often involve more sensitive information, such as biometric data, which can be used to identify individuals [44]. This means that data used in AI are often associated with more significant privacy concerns than administrative data, such as birth and death registries. Our study is the first to address specific factors related to data sharing for AI in LMICs, which is of critical importance given AI’s potential to enable developing health systems to leapfrog global health inequalities [45].

The most relevant factors we identified were technical and infrastructural, policy based, or motivational. Many of the factors we identified in our review were also seen in 2 previous studies focusing on HIE adoption and barriers to sharing research and scientific data [10,11]. For example, van Panhuis et al [13] found that similar technical factors, such as lack of data standards, guidelines, and policies, were important barriers to sharing data in public health. In our study, infrastructural factors, such as limited access to information and communications technology, unreliable internet connectivity, frequent power shortages, equipment deficiencies, and software instability, were important factors hindering data storage and management. The unavailability of appropriate software and the persistence of handwritten records (often in regional languages) were particular challenges to data integration and sharing efforts. These findings were consistent with previous literature [46-49] and are also seen when data are shared for reasons other than AI, such as global health emergencies and disease outbreaks [50,51]. We found that limited data digitization is an important upstream barrier to downstream data sharing in LMICs. Furthermore, barriers at the political and policy level were significant limiting factors to data sharing for AI in health care. Previous studies have emphasized the need for data-sharing governance mechanisms to protect vulnerable populations in the global south [52-54]. Our results confirm earlier studies and suggest that developing clear policies and closing regulatory gaps can promote data sharing [10,46,48,55]. The motivational barriers we identified, including poor staff and management motivation, confirm previous results [56]. Public and staff education, a clear data-sharing incentive structure, and user-friendly software could improve staff motivation [48,57].

Many barriers identified in the systematic review were relevant to the Thai context. For example, limited staff time to collect data, a limited understanding of data’s value, and low health literacy were important barriers in Thailand and the systematic review. Aljunid et al [47] previously reported that Thailand’s fragmented financing schemes and service provision limit stakeholder cooperation in data sharing. Our findings concur that this health system fragmentation may hinder clinical and management staff from sharing data. Furthermore, stakeholders pointed to a lack of software standardization within the same organization and continued reliance on manual data management and paper-based EHRs. These findings differ from a previous qualitative study [58], where Thai stakeholders did not see technical challenges as a primary barrier to health care data sharing and instead placed greater weight on ethical concerns, such as informed consent and data control. However, Thai-specific literature [47,54] supports our findings that fragmented databases and software unavailability limit local health data sharing. Finally, we found that unclear data-sharing regulations and guidelines in Thailand, that is, complex and unclear policies and low applicability of international data standards—prevent health care stakeholders from sharing data. These findings are supported by earlier studies, emphasizing health data sharing in Thailand echoes the importance of establishing clear data management and sharing policies [54,55].

During interviews, stakeholders suggested several improvements to overcome barriers to sharing data. First, building a conducive digital ecosystem and having shared data input platforms for health facilities can ensure data uniformity and compatibility. Currently, similar initiatives are being undertaken by the Thai National Health Information Standards Development Centre under the Health System Research Institute of the Ministry of Public Health, Thailand. This organization develops and promotes national standards for health information systems, including data elements, codes, and terminologies, to ensure interoperability across health care organizations [59]. Singapore’s National Electronic Health Record initiative is also an example of creating a unified health information system by standardizing data formats and terminologies for seamless data exchange across health care providers [60]. Second, developing predefined templates, easy-to-understand consent forms, and standardized guidelines for data sharing, intellectual property rights, and compensation for data breach victims can help Thailand overcome social-, ethical-, political-, and policy-level barriers at various levels. These measures will increase data owners’ and health care professionals’ awareness of data sharing and prevent misunderstandings of data sharing guidelines such as the PDPA. In addition, these steps will help developers create and validate transparent and mutually trustworthy data-sharing models across health facilities. Singapore’s Advisory Guidelines for Key Concepts on PDPA can serve as a framework for Thailand, offering detailed guidance and examples to understand and interpret key terms and obligations in the PDPA [61]. The document provides illustrative examples of important PDPA terms, including what qualifies as “personal data,” the concept of “controlling and owning one’s personal data,” and the “roles and obligations of data intermediaries.” Third, training health care professionals such as hospital administrators, doctors, and care providers can improve the effectiveness of the aforementioned measures. Access to improved digital infrastructures and adequate training on cybersecurity safeguards will enable efficient and standardized data sharing.

### Limitations

Although the study focused on a specific context, we have provided detailed descriptions of the settings and participants to allow readers to judge the applicability of the findings to other contexts. Studies included in our systematic review output were mostly from Africa and Asia. Therefore, results might not be generalizable to other regions. LMICs can use this study as a framework and leverage their individual health systems’ barriers and strengths to devise local solutions for enhanced data sharing. While the classification of barriers and enablers across domains was done objectively and cross-checked by multiple authors, some factors identified were relevant to various framework categories. This is inevitable due to the complexity of health information systems, the various stakeholders involved, and their interrelationships. Furthermore, the response rate for our stakeholder interviews was low, reducing the sample’s representativeness.

### Conclusions

AI has the potential for LMICs to leapfrog health inequalities and deficiencies in health systems. Our review identified significant barriers to sharing data for AI according to a comprehensive framework. In a localized case study, most barriers from the systematic review were relevant to the Thai context. In Thailand, data architecture, inconsistent data standards, complex and unclear policies, uneven distribution of financial resources across institutions, and confidentiality breaches were reported as important barriers to data sharing for AI. Together, our results provide insight into the challenges that LMICs’ health systems face regarding data sharing for AI.

## Appendix

Multimedia Appendix 1 Search strategy for different databases.

Multimedia Appendix 2 Critical appraisal of qualitative studies using Critical Appraisal Skills Programme checklist.

Multimedia Appendix 3 Interview guide—barriers and enablers to data sharing in digital health in Thailand.

Multimedia Appendix 4 PRISMA checklist.

## Abbreviations

AI: artificial intelligence
EHR: electronic health record
EMR: electronic medical record
HIE: health information exchange
LMICs: low- and middle-income countries
ML: machine learning
PDPA: Personal Data Protection Act
SPICE: settings, perspective, intervention, comparison, and evaluation

## Data Availability

The datasets generated during and analyzed during this study are available from the corresponding author on reasonable request.
