# Supplementary material for: Challenges and Opportunities for Data Sharing Related to Artificial Intelligence Tools in Health Care in Low- and Middle-Income Countries: Systematic Review and Case Study From Thailand
Source: J Med Internet Res. 2025 Feb 4;27:e58338. doi: 10.2196/58338 (PMC11836587; doi:10.2196/58338)
Supplement: Multimedia Appendix 1 [file jmir_v27i1e58338_app1.pdf]

APPENDIX 1: SEARCH STRATEGY FOR DIFFERENT DATABASES.

		**Concept 1 /Population/Problem**	**Concept2 /Intervention/Exposure**	**Concept2 /Intervention/Exposure**	**Additional concept (if any) /comparison/outcome**
**Key concepts**<br><br>*Identify the key concepts based on your research topic.*		Data sharing	AI tools	Healthcare domain	Barriers and enablers
**Free text terms / natural language terms**<br><br>(synonyms, UK/US terminology, medical/laymen's terms, acronyms/abbreviations, drug brands, more narrow search terms)<br><br>*List down your keywords for each concept.*		Data sharing or data exchange or information exchange or information sharing or data transfer or data transmission OR information distribution OR information dissemination	Artificial intelligence or computational intelligence or distributed intelligence or artificial systems or expert system or decision support or Electronic health record or machine learning or machine intelligence or AI or deep learning or intelligent system or computer vision system	Healthcare or health care or health service or health industry or health sector	Barrier or enabler or challenge or facilitator or impediment or obstacle or roadblock or opportunity
**PubMed**	**Controlled vocabulary terms**<br><br>MeSH terms	Data sharing	Artificial intelligence	Healthcare, health service	
	**Keyword statement**	"Information Dissemination"[Mesh]<br><br>Keyword<br><br>Data sharing*[Title/Abstract] OR data exchang*[Title/Abstrac	"Artificial Intelligence"[Mesh]<br><br>Keyword<br><br>Artificial intelligence[Title/Abstract] OR computational	"Health Services"[Mesh] OR "Health Care Sector"[Mesh]<br><br>Keyword<br><br>Healthcare[Title/Abstract] OR health	Keyword<br><br>barrier*[Title/Abstract] OR enabler*[Title/Abstract] OR challenge*[Title/Abstract] OR opportunit*[Title/Abstract] OR impediment*[Title/A

		t] OR information exchang*[Title/Abstract] OR information sharing*[Title/Abstract] OR data transfer*[Title/Abstract] OR data transmission*[Title/Abstract] OR information distribution*[Title/Abstract] OR information dissemination*[Title/Abstract]	intelligence[Title/Abstract] OR distributed intelligence[Title/Abstract] OR artificial system*[Title/Abstract] OR expert system*[Title/Abstract] OR decision support [Title/Abstract] OR Electronic health record*[Title/Abstract] OR machine learning[Title/Abstract] OR machine intelligence[Title/Abstract] OR AI[Title/Abstract] OR deep learning[Title/Abstract] or intelligent system*[Title/Abstract] OR computer vision system*[Title/Abstract]	care[Title/Abstract] OR health service*[Title/Abstract] OR health industr*[Title/Abstract] OR health sector*[Title/Abstract]	bstract] OR obstacle*[Title/Abstract] OR facilitator*[Title/Abstract] OR roadblock*[Title/Abstract]
**Scopus**	**Keyword statement**	TITLE-ABS-KEY ("data sharing" OR "data exchange" OR "information exchange" OR "information sharing" OR "data transfer" OR "data transmission" OR “information distribution” OR “information dissemination”)	TITLE-ABS-KEY ("artificial intelligence" OR “computational intelligence” OR "distributed intelligence" OR "artificial system*" OR "expert system*" OR "decision support" OR "electronic health record*" OR "machine learning" OR "machine intelligence" OR “AI” OR "deep learning" OR "intelligent system*" OR “computer vision system*”)	TITLE-ABS-KEY ("healthcare" OR "health care" OR "health service" OR “health industry” OR “health sector”)	TITLE-ABS-KEY ("enabler*" OR "barrier*" OR "challenge*" OR “facilitator*” OR “impediment*” OR “obstacle*” OR “roadblock*” OR “opportunit*”)

**Web of Science**	**Keyword statement**	TS = ("data sharing*" OR "data exchange*" OR "information exchange*" OR "information sharing*" OR "data transfer*" OR "data transmission*" OR "information distribution*" OR "information dissemination*")	TS=("artificial intelligence" OR "computational intelligence" OR "distributed intelligence" OR "artificial system*" OR "expert system*" OR "decision support" OR "electronic health record*" OR "machine learning" OR "machine intelligence" OR "AI" OR "deep learning" OR "intelligent system*" OR "computer vision system*")	TS=("healthcare" OR "health care" OR "health service*" OR "health industry*" OR health sector*")	TS=("enabler*" OR "barrier*" OR "challenge*" OR "facilitator*" OR "impediment*" OR "obstacle*" OR "roadblock*" OR "opportunit*")
**Emtree.com**	**Controlled vocabulary terms**		Artificial intelligence	Healthcare, health service	
	**Keyword statement**	<u>Keyword</u><br><br>('Data sharing*' OR 'data exchang*' OR 'information exchang*' OR 'information sharing*' OR 'data transfer*' OR 'data transmission*' OR 'information distribution*' OR 'information dissemination*'):ab,ti	"Artificial Intelligence"<br><br><u>Keyword</u><br><br>('artificial intelligence' OR 'computational intelligence' OR 'distributed intelligence' OR 'artificial system*' OR 'expert system*' OR 'decision support' OR 'electronic health record*' OR 'machine learning' OR 'machine intelligence' OR 'ai' OR 'deep learning*' OR 'intelligent system*' OR	"Health Service"<br><br><u>Keyword</u><br><br>'healthcare':ab,ti OR 'health care':ab,ti OR 'health service*':ab,ti OR 'health sector*':ab,ti OR 'health industr*':ab,ti	<u>Keyword</u><br><br>'barrier*':ab,ti OR 'enabler*':ab,ti OR 'challenge*':ab,ti OR 'opportunit*':ab,ti OR 'facilitator*':ab,ti OR 'impediment*':ab,ti OR 'obstacle*':ab,ti OR 'roadblock*':ab,ti

			'computer vision system*'):ab,ti		
**ACM**	**Keyword statement**	AllField:("data sharing*" OR "data exchange*" OR "information exchange*" OR "information sharing*" OR "data transfer*" OR "data transmission*" OR "information distribution*" OR "information dissemination*")	AllField:("artificial intelligence" OR "computational intelligence" OR "distributed intelligence" OR "artificial system*" OR "expert system*" OR "decision support" OR "electronic health record*" OR "machine learning" OR "machine intelligence" OR "AI" OR "deep learning" OR "intelligent system*" OR "computer vision system*")	AllField:("healthcare" OR "health care" OR "health service*" OR "health industr*" OR "health sector*")	AllField:("enabler*" OR "barrier*" OR "challenge*" OR "facilitator*" OR "impediment*" OR "obstacle*" OR "roadblock*" OR "opportunit*")
