# Supplementary material for: Challenges and Opportunities for Data Sharing Related to Artificial Intelligence Tools in Health Care in Low- and Middle-Income Countries: Systematic Review and Case Study From Thailand
Source: J Med Internet Res. 2025 Feb 4;27:e58338. doi: 10.2196/58338 (PMC11836587; doi:10.2196/58338)
Supplement: Multimedia Appendix 2 [file jmir_v27i1e58338_app2.docx]

# Appendix 2 Critical appraisal of qualitative studies using CASP checklist.

| Study | Clear statement of the aims of the research? | Qualitative methodology appropriate? | Research design appropriate? | Recruitment strategy appropriate? | Data collected in a way that addressed the research issue? | Relationship between researcher and participants adequately considered? | Ethical issues considered? | Data analysis sufficiently rigorous? | Clear statement of findings? | How valuable is the research? |
| --- | --- | --- | --- | --- | --- | --- | --- | --- | --- | --- |
| Walcott-Bryant, A. [35] | Y | Y | Y | Y | Y | Y | CT | Y | Y | Y |
|  | Y | Y | Y | Y | Y | Y | CT | Y | Y | Y |
| Chukwu, E. [1] | Y | Y | Y | Y | Y | CT | CT | Y | Y | Y |
|  | Y | Y | Y | Y | Y | CT | CT | Y | Y | Y |
| De Fatima Marin, H. [31] | Y | Y | Y | Y | Y | CT | CT | Y | Y | Y |
|  | Y | Y | Y | Y | Y | CT | CT | Y | Y | Y |
| Ntlhakana, L. [32] | Y | Y | Y | Y | Y | CT | Y | CT | Y | Y |
|  | Y | Y | Y | Y | Y | CT | Y | CT | Y | Y |
| Pradhan, K. [3] | Y | Y | CT | CT | CT | Y | Y | CT | Y | Y |
|  | Y | Y | CT | CT | CT | Y | Y | CT | Y | Y |
| Bhattacharya, S. [25] | Y | Y | Y | CT | CT | CT | CT | CT | Y | Y |
|  | Y | Y | Y | CT | CT | CT | CT | CT | Y | Y |
| Troncoso, E. L. [30] | Y | Y | Y | Y | CT | CT | CT | CT | Y | Y |
|  | Y | Y | Y | Y | CT | CT | CT | CT | Y | Y |
| Akhlaq, A. [38] | Y | Y | Y | Y | Y | Y | Y | Y | Y | Y |
|  | Y | Y | Y | Y | Y | Y | Y | Y | Y | Y |
| Muinga, N. [39] | Y | Y | Y | Y | Y | Y | Y | Y | Y | Y |
|  | Y | Y | Y | Y | Y | Y | Y | Y | Y | Y |
| Wang, Z. [33] | Y | Y | Y | Y | Y | CT | N | CT | Y | Y |
|  | Y | Y | Y | Y | Y | CT | N | CT | Y | Y |
| Deriel, E. [34] | Y | Y | Y | Y | Y | CT | Y | Y | Y | Y |
|  | Y | Y | Y | Y | Y | CT | Y | Y | Y | Y |
| Chali, F. [2] | Y | Y | Y | CT | N | CT | Y | CT | Y | Y |
|  | Y | Y | Y | CT | N | CT | Y | CT | Y | Y |
| Yaqoob, T. [9] | Y | Y | Y | Y | Y | Y | Y | CT | Y | Y |
|  | Y | Y | Y | Y | Y | Y | Y | CT | Y | Y |
| Lei, J. [26] | Y | Y | Y | CT | Y | N | N | CT | Y | Y |
|  | Y | Y | Y | CT | Y | N | N | CT | Y | Y |
| Ndlovu, K. [27] | Y | Y | Y | CT | CT | Y | Y | CT | Y | Y |
|  | Y | Y | Y | CT | CT | Y | Y | CT | Y | Y |
| Keny, A. [36] | Y | Y | Y | Y | Y | Y | CT | Y | Y | Y |
|  | Y | Y | Y | Y | Y | Y | CT | Y | Y | Y |
| Simbini, T. [28] | Y | Y | Y | CT | Y | Y | Y | CT | Y | Y |
|  | Y | Y | Y | CT | Y | Y | Y | CT | Y | Y |
| Nutley, T. [37] | Y | Y | Y | Y | Y | Y | CT | CT | Y | Y |
|  | Y | Y | Y | Y | Y | Y | CT | CT | Y | Y |
| Shrivastava, Swapnil [29] | Y | Y | Y | CT | CT | CT | CT | CT | Y | Y |
|  | Y | Y | Y | CT | CT | CT | CT | CT | Y | Y |
| Fakhkhari, Houda [6] | Y | Y | Y | Y | Y | N | N | Y | Y | Y |
|  | Y | Y | Y | Y | Y | N | N | Y | Y | Y |
| Botha, Marna [7] | Y | Y | Y | Y | Y | Y | Y | Y | Y | Y |
|  | Y | Y | Y | Y | Y | Y | Y | Y | Y | Y |
| Guidry, Alicia F. [8] | Y | Y | Y | Y | Y | Y | Y | Y | Y | Y |
|  | Y | Y | Y | Y | Y | Y | Y | Y | Y | Y |
