# Supplementary material for: Challenges and Opportunities for Data Sharing Related to Artificial Intelligence Tools in Health Care in Low- and Middle-Income Countries: Systematic Review and Case Study From Thailand
Source: J Med Internet Res. 2025 Feb 4;27:e58338. doi: 10.2196/58338 (PMC11836587; doi:10.2196/58338)
Supplement: Multimedia Appendix 3 [file jmir_v27i1e58338_app3.pdf]

APPENDIX 3: INTERVIEW GUIDE— BARRIERS AND ENABLERS TO DATA SHARING IN DIGITAL HEALTH IN THAILAND.

1. What are your roles/ your organization's roles in supporting responsible data sharing in digital health?
2. What are different barriers to data sharing in digital health? Subsequently, what are the enabling forces implemented to overcome these barriers?
   - Economic barriers
   - Ethical barriers
   - Political, legal and policy barriers
   - Technical barriers
   - Data barriers
   - Motivational barriers
   - Social barriers
   - Organisational and managerial barriers
3. What are some of the strategies taken to build a conducive digital ecosystem that facilitates responsible data sharing in digital health?
4. What are your suggestions/recommendations for other countries aspiring to improve governance and organisational practices to facilitate responsible data sharing?

Last question/Snowballing:

Anybody else, you might know that we can talk to/has expertise in this area?
